# Treatment outcomes in locally advanced colorectal carcinoma

**DOI:** 10.1186/1477-7800-1-8

**Published:** 2004-11-04

**Authors:** K Harish, YV Narayanaswamy, S Nirmala

**Affiliations:** 1 Department of Surgical Oncology, M. S. Ramaiah Medical College & Hospital, Bangalore – 560054, India; 2 Department of General Surgery, M. S. Ramaiah Medical College & Hospital, Bangalore – 560054, India; 3 Department of Radiation Oncology, M. S. Ramaiah Medical College & Hospital, Bangalore – 560054, India

## Abstract

**Background:**

Locally advanced colorectal cancers form a distinct subgroup where contiguous organs could be involved without distant metastases and so may be amenable to curative surgical resection. It was our objective to report our experience in treating six such patients with operable locally advanced colorectal carcinomas.

**Methods:**

We retrospectively reviewed the case notes of 47 patients who were diagnosed with colorectal cancers at M S Ramaiah Medical Teaching Hospital between the years 1996 – 2001. Six patients were identified with T4 lesions, adjacent organ involvement and with no nodal involvement. The treatments and outcomes for these patients were then reviewed.

**Results:**

Two of three patients with rectal malignancies who underwent pelvic exenteration succumbed to disease recurrence within the first 18 months. One of the three patients with colonic cancers died of non malignant causes. The other two are disease free till date.

**Conclusions:**

Aggressive multivisceral resections for locally advanced colonic cancers might be appropriate. Rectal cancers when locally advanced may be considered for pelvic exenteration, but a more guarded prognosis may apply.

## Background

Locally advanced colorectal tumors constitutes to about 5 – 22% of all colorectal cancers at the time of presentation [1]. This type of tumor forms a distinct sub class of colorectal tumors characterized by aggressive local behavior in the form of invasion of adjacent organs or structures with somewhat surprisingly no distant metastasis at presentation. The survivals of such cases that undergo multivisceral resections are 58% and 43% for UICC stage II and III respectively. These results are similar to those undergoing conventional resections [1]. In addition, there is a suggestion of elevated stage related late results to the same level as that associated with tumors where there is no direct invasion of contiguous organs [1]. Addressing the local disease adequately with multi-visceral resections when necessary could result in favorable out come. However en-bloc surgical resection of the tumor forms a surgical challenge and the risks of complications and death must be weighed against probable survival benefits. The post operative complication rate of multivisceral resection is about 11.5%; 30 day operative mortality is 3.6% and compares favorably with non-multivisceral resections [1]. It was our objective to report our experience in treating six such patients with operable locally advanced colorectal carcinomas.

## Methods

During the years 1996–2001, 47 patients with colorectal cancers were treated at our institute. All patients underwent a rectal exam, punch biopsy if it were an accessible rectal lesion or colonoscopy and biopsy if it were a colonic lesion. Screening colonoscopy for a synchronous lesion was however done for all patients. Patients in whom a biopsy confirmed a malignant lesion underwent an ultrasound (US) study of the abdomen, chest X-ray and Carcino-embryonic antigen (CEA) estimation. Patients with doubtful involvement of adjacent organs or structures underwent CT scan. Cystoscopy was performed when urinary bladder involvement was suspected on CT scan. Patients were diagnosed as locally advanced when the staging evaluation showed involvement of adjacent organ or structure. Though nodal evaluation is suboptimal with US or CT scan, all patients with 'N0' status on these investigations were included as locally advanced while those with 'N+' on evaluation were excluded. Patients with metastasis to any organ were excluded. The preoperative staging of the tumor was T4, N0, M0. Elective surgery was performed on all cases. No pre-operative therapy was administered. All patients were administered adjuvant radiation and chemotherapy which was instituted immediately after wound healing (between 16 and 29 days post surgery). A total dose of 5000 centi-Gray of external beam radiation in combination with chemotherapy consisting of 5-Fluoro-Uracil (5-FU) and leucovorin was administered. The dose of 5-FU was 425 mg/m^2 ^and that of leucovorin 20 mg/m^2^. This combination was administered as infusion on day 1 to 5, and six such cycles every 28 days were instituted as part of chemotherapy regimen. The therapy in colonic carcinoma was sequenced as 1 cycle of chemotherapy followed by radiation and the remaining 5 cycles after completion of radiation. In case of rectal cancers, the chemotherapy was administered concurrently with radiation. The administration of leucovorin was with-held during radiation and instead, the dose of 5-FU was increased to 500 mg/m^2^. Follow up was by clinical examination every 3 months for first two years, and six monthly until 5 years. The evaluation included alternate year CT scan, annual US abdomen, chest X-ray, colonoscopy and CEA levels every six months.

The two live patients have given consent for publication. Consents have been obtained from the legal heirs of the remaining four patients.

## Results

The study group consisted of six patients (29 to 70 years), three of whom had locally advanced colonic disease and three of whom had locally advanced rectal disease. Pre operative CEA levels were surprisingly within normal limits in all six patients. The pre operative colonoscopic or rectal biopsy had determined adenocarcinoma in all six patients (Grade as shown in Table 1). In two of the patients with rectal disease where involvement of the bladder wall was suspected, cystoscopic findings were normal. All the patients were offered radical surgery on elective basis. The radicality included removal of adjacent involved organs which could increase the complication rate of extensive surgery. In addition such extended resections for rectal cancer would result in exenterative pelvic surgery resulting in abdominal stomas. Surgical resection margins were negative in all cases.

**Table 1:** Salient Clinico – pathologic features of locally advanced colorectal carcinomas

**Site of malignancy**	**Age Sex**	**Pathologic 'T' Status**	**Grade**	M/SR ^* ^ca	**Adjacent ** **Org ^†^**	**Nodal Status**	**PNS ^‡^**	**PN/PV ^§ ^spread**
**Colon**	56 Male	T 4	II	-	+ (abdominal wall)	N0	-	-
	58 Male	T4	II	-	+ (abdominal wall)	N0	-	+
	67 Female	T3	II	-	- (bladder dome)	N0	-	-
**Rectum**	45 Male	T4	I	+	+ (bladder / prostate)	N1	+	+
	27 Female	T4	I	+	+ (uterus and vagina)	N2	+	+
	69 Male	T4	II	+	+ (bladder / prostate)	N2	+	+

* Mucinous or signet ring carcinoma

**† **Pathologic involvement of adjacent organ

**‡ **Perinodal spread

**§ **Perineural / perivascular spread

### Case 1

**Figure 1:**
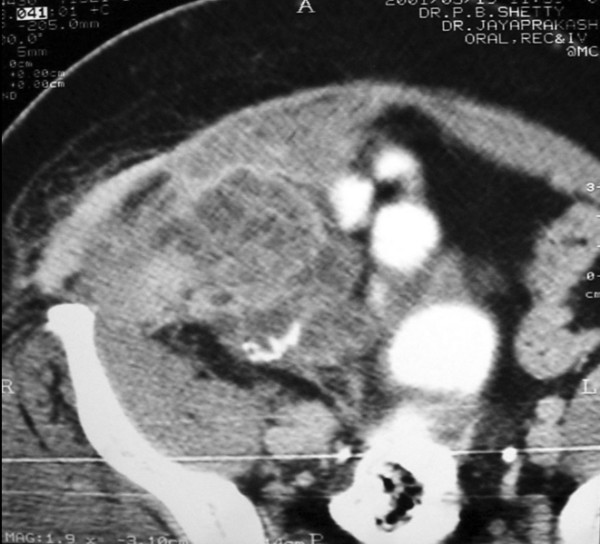
CT scan of the abdomen showing the involvement of abdominal wall muscles and adhesions to neighboring intestines.

**Figure 2:**
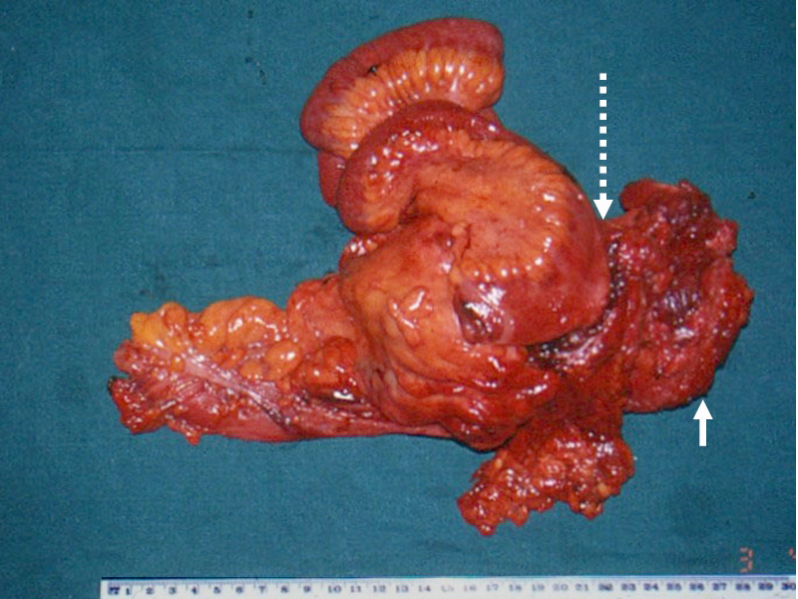
Specimen of radical right hemicolectomy with en-bloc resection of abdominal wall muscle (indicated by small arrow) and neighboring small intestines (indicated by dotted arrows).

Patient presented with a 2 month history of abdominal mass. There was no history of weight loss or altered bowel habits. The mass was firm with irregular borders and restricted mobility. CT scan of abdomen revealed a large mass lesion arising from the caecum and ascending colon apparently infiltrating the anterior abdominal wall (Fig 1). Patient underwent a radical right hemicolectomy with en-bloc resection of involved abdominal wall (Fig 2). The right branch of the middle colic, the right colic and ileocolic vessels were ligated at the origin. Retroperitoneum was not involved. A two layer hand sewn ileocolic anastomosis was performed. The abdominal wall muscles were resected with a 2.5 cm margin and the resultant defect was repaired with a polypropylene mesh. The patient received adjuvant radiation and chemotherapy as described above and is disease free 3 1/2 years after surgery.

### Case 2

Patient presented with a 6 month history of bleeding per rectum. There was no history of weight loss. Patient's hemoglobin was 10.2 gms / dl. Patient had ignored the complaint for 3 months but even at a later date was unfortunately not advised to undergo sigmoidoscopy where he was evaluated. Patient underwent colonoscopic evaluation at our institute after evaluation. CT scan showed possible involvement of abdominal wall with the growth arising from sigmoid colon. Intra-operatively, the mass was found to involve the posterior rectus sheath. Sigmoid colectomy was performed along with resection of the involved posterior rectus sheath and the rectus muscle en-bloc. Splenic flexure was mobilized to obtain colorectal hand sewn anastomosis. Primary closure of the rectus defect was achieved and patient received adjuvant radiation and chemotherapy. The patient is disease free 3 years post surgery.

### Case 3

Patient was diabetic, asymptomatic; ultrasound performed for evaluation of kidneys for diabetic nephropathy incidentally detected the mass lesion in sigmoid colon. Biopsy specimen was obtained at colonoscopy; no synchronous lesion was detected in the rest of the colon. CT scan revealed close relation of the sigmoid mass lesion to the bladder dome but was unable to comment categorically on wall infiltration. Urinary bladder mucosa was intact and normal on pre-operative cystoscopy. Intra-operatively, the lesion was found adherent to bladder dome. Sigmoid colectomy and part of the bladder wall was resected en-bloc. There was no pathologic evidence of bladder involvement. Patient had ishcaemic heart disease and diabetic nephropathy; she later developed myocardial infarction and renal failure while on adjuvant therapy 7 months post surgery and succumbed to the same.

### Case 4

Patient had bleeding per rectum and constipation for 18 months. Patient was evaluated and treated as hemorrhoids initially (no surgical intervention). About 6 months prior to presentation at our institute, he was diagnosed as rectal cancer and was advised abdomino-perineal resection. Patient was scared of surgery and waited without any therapy. Rest of the large bowel was normal on colonoscopy. A CT scan showed involvement of the urinary bladder and prostate by a rectal mass while bony pelvis appeared free of tumor involvement. The patient underwent a total pelvic exenteration with formation of a urinary diversion by ileal conduit. After a lower midline laparotomy, abdomen was evaluated for any ascites or liver metastasis. Para-aortic area was palpated and rest of peritoneal cavity was evaluated for any metastatic deposit. Inferior mesenteric artery was ligated beyond the origin of the left colic artery. Bilateral pelvic nodal dissection was completed. Sigmoid colon and rectum was mobilized as in abdominoperineal resection over the sacral hollow. Anteriorly the dissection was carried out in the retropubic space to access the urethra beyond the prostate. Lateral dissection was carried out which included the ligation of the superior vesical vessels. Both the ureters were ligated below the pelvic brim in the true pelvis at least 3 cm proximal to palpable disease. Isolated loop of ileum was mobilized based on two vessels. One end of the loop was closed and both ureters were anastomosed separately to the loop. Intestinal continuity was obtained with ileo-ileal end to end anastomosis. This ileal loop was brought out as a urinary stoma on the right side and the cut colon was brought out as colostomy on the left side. The patient's postoperative recovery was unremarkable except for a uereteric leak which settled by 10^th ^post operative day. Patient received adjuvant therapy but was irregular on follow-up and refused to be investigated. Patient developed hepatic metastasis and succumbed to the disease after 18 months.

### Case 5

Patient had bleeding per rectum and constipation for 4 months. Patient had significant weight loss over previous 2 months. Patient had circumferential rectal carcinoma involving uterus and upper part of vagina. Rectal carcinoma was proved by biopsy. Rest of the colon was normal on colonoscopy. Upper vaginal mucosa was intact but indurated underneath. CT scan showed infiltration of lower part of uterus and upper vagina. Patient underwent posterior pelvic exenteration. Uterus, including both the fallopian tubes and ovaries were removed en-bloc along with vagina and rectum. Surgery was followed by adjuvant radiation and chemotherapy. Patient succumbed to the disease 1 year later with multiple metastases in liver, brain and malignant ascites.

### Case 6

**Figure 3:**
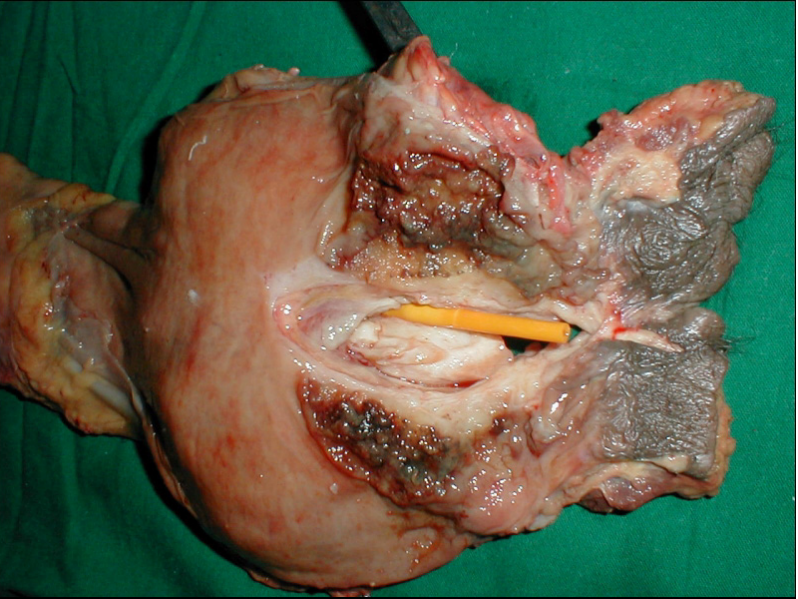
Specimen of pelvic exenteration with cut open rectum showing the rectal adenocarcinoma infiltrating the prostate. Foley's catheter is also seen.

Patient had bleeding per rectum and constipation for 3 months. A preoperative CT scan with rectal and intravenous contrast revealed involvement of prostate and possibly bladder. Colonoscopy did not reveal any other lesion. He underwent a total pelvic exenteration (Fig 3) with ileal conduit similar to case 4. Patient developed fever 48 hours after surgery. Evaluation for infective pathology including cultures from catheters and venous access tips did not reveal any source of infection. There was no pocket of collection on repeated abdominal ultrasound. There was raised leukocyte count. A possibility of sepsis from an occult focus was considered and treated. However, on the 5^th ^post operative day, patient developed vomiting and had raised serum creatinine values. Values of fibrin degradation products were also raised and a diagnosis of disseminated intravascular coagulation and renal failure was entertained. Patient had prolonged prothrombin time but did not have any clinical bleed. He died on 30^th ^day post operative while on recovery from the same. The post surgical pathology reports are summarized in Table 1.

## Discussion

Surgical resection remains the primary and most effective treatment for advanced colorectal cancers [2]. The 5-year survival rate of the overall group of patients undergoing multivisceral resection is 42%, that of the subgroup undergoing curative surgery is 51%, and that of the subgroup receiving only palliative resection is 0% [1]. In the presence of local tumor extension the distinction between inflammatory adherence and malignant invasion is impossible to make intra operatively. Adherence of the neoplasm to surrounding structures demonstrates pathological tumor invasion in approximately 50% of cases [1,2]. The operative intent of the surgeon should be to achieve complete extirpation with adequate margins in the involved structures. Dissection between malignancy and adherent structures, or biopsy and frozen section is not recommended as these procedures may promote tumor dissemination [1,2], which may have a detrimental impact on prognosis [3-5]. The concept of en-bloc resection has significantly reduced the local recurrence rate from 77% to 36% and significantly improved the 5 year over all survival of 43% [1,4-6]. Other studies have also shown improved survival of similar staged colorectal cancers with en-bloc resection [7,8]. The survivals reported in various studies are shown in Table 2[1,2,4,9-11]. The 5-year survivals are comparable ranging from 38% to 52%. Although colon and rectal cancers which required multivisceral resections are shown separately, the survivals are not indicated differently. For similar 'T' status, the corresponding 'N+' status of colon versus rectum has not been studied. Our present study is small but the overall survival is 33% and compares with larger studies. In addition, since a very high percentage of even large T4 tumors have not yet metastasized to the regional lymph nodes, a multivisceral resection offers the chance to radically remove the local disease and affect a cure [1,12]. All patients in our study group underwent en-bloc resection. No attempt was made to separate the adherent structure to confirm or negate the involvement of adjacent organ per operatively. Since colon spans the entire periphery of the abdomen, almost all the abdominal organs have been reported to be involved [2]. Multivisceral resections have been recommended whenever necessary as it could offer cure or at least significant palliation [13-15]. En-bloc resections have been recommended even when it warrants a pancreatico-duodencetomy [16,17]. In the present report, the en-bloc resections included abdominal wall in two cases and urinary bladder in one.

**Table 2:** Summary of survival data from a few large studies

**Author**	**Multivisceral Resections**	**Colon**	**Rectum**	**Death Rate**	**5-year Survival Rate**
Reiner (1987)	158	53	105	12%	38%
Heslov (1988)	58	26	32	5%	38%
Montesani (1991)	33	-	-	-	30%
Hermanek (1992)	197	119	78	3%	52%
Gebhardt (1999)	173	122	51	4%	51%
Lehnert (2002)	201	139	62	7.5%	51%

Rectum cancers involve the pelvic genital organs and or urinary bladder anteriorly. Posteriorly it could involve the sacrum. Exenterative pelvic surgeries are warranted for locally advanced rectal cancers [18]. Pelvic exenteration and sacral resection for primary or recurrent rectal cancer are tolerable procedures with a low mortality rate [19]. Although they provide a survival benefit if curative resection is possible, the associated morbidity remains high [19-21]. The complications described include sepsis, intra-abdominal abscess, pelvic abscess, enteric fistula, enteric anastomotic leak, wound infection, ileoureteral anastomotic leak, ileoureteral anastomotic stenosis, bowel obstruction, vascular injury, bleed, liver dysfunction and pneumonia. In the present study, both the male patients underwent total pelvic exenteration with ileal conduit for urinary diversion. The female patient underwent posterior pelvic exenteration. The male patient who lived for 18 months did not have any major problem to take care of both the stomas. Similar thoughts that a small reduction in patient's quality of life due to urinary diversion should not be a major contraindication when surgery with urinary diversion is warranted to obtain curative resection have been echoed [22].

Pre-operative nodal evaluation by abdominal CT scan, MRI or endoscopic ultrasound may be inadequate. Though endosonography can detect perirectal nodes, its inability to reliably predict pathologic involvement is a constraint [23]. Nodal status assessment is considered adequate when at least 14 nodes are examined [24]. In some studies, nodal metastasis has insignificantly altered survival [4,6,25,26]. In contrast, other studies have shown that presence of lymph node involvement is associated with poor prognosis [21]. Studies have shown that 5 year survival in patients with nodal metastasis was 0% to 11%, significantly lower than the 37% to 76% survival rate in their patients without nodal metastases [11,27,28]. These studies have even cast doubts over usefulness of pelvic exenteration in patients with nodal disease though some would still recommend it [29]. None of the patients in the study were evaluated by endosonography but were evaluated by CT scan. Accepting that it is a poor tool for nodal evaluation, pre-operative involvement was not detected in any patient in the study group. Moreover, lymph node status can be accurately determined by pathologic examination only. Hence the investigations might not contribute to decision making though cases with obvious metastasis could be excluded from major resections. In our series of six cases, all the colonic patients were node negative and had no vascular invasion. With multi visceral resections, T4 colonic cancers had acceptable morbidity and better treatment outcome. However, rectal cancer patients had poor prognostic factors like vascular invasion, lymph nodal involvement and histological type of mucinous / signet ring variety. Another striking feature was that though colonic cancers were grade II tumors, they were pathologically N0. But rectal cancers were pathologically N1, N2 and had perinodal spread despite being grade I tumors.

Down staging of locally advanced rectal cancers have been achieved with pre-operative radiation or chemotherapy or both resulting in decreased recurrence and improved disease free survival [30-35]. The results of IORT hold some promise [36,37]. It would be fair to say that there is still no agreement as to the optimal sequencing of chemotherapy, radiation, and definitive surgery in immediately operable patients, and both preoperative and postoperative approaches have vocal proponents [38]. In the present study, pre-operative chemotherapy or radiation was not administered to any patient. There have been no studies to suggest that pre-operative chemoradiation would decrease the magnitude of surgical resection and hence a patient suitable for exenteration would still require the same after such a therapy. In addition, administration of radiation pre-operatively increases the morbidity after exenteration [39]. It must also be noted that studies on pre-operative chemoradiation have shown improvement is DFS and not in OS. Till such time there are conclusive results in these areas for pre-operative therapy with chemoradiation, such a therapy would continue to be debated.

Although it could be inappropriate to draw conclusions from a small number of cases, certain features require further deliberation. T4 colonic cancers have had better outcomes compared to T4 rectal cancers. Many of the earlier reports have combined results of colonic and rectal cancers. In addition, the reports have combined locally advanced and recurrent cancers. As the tumor biology of these areas is different, the results of colon and rectum have to be viewed separately. In addition, the morbidity and mortality associated with multivisceral resections are different of these areas. Since nodal metastasis were seen in all T4 rectal cancers and in none of the colonic T4 cancers, it would be interesting to evaluate percentage of nodal metastasis separately for colonic and rectal cancers with similar T stages. Another indicator of aggressive biology of rectal cancers compared to colonic cancers is the fact that lower grade rectal cancers had more nodal metastasis, perineural and perivascular invasion compared to higher grade colon cancers.

## Conclusions

Locally advanced colonic cancers are to be evaluated and treated aggressively with multivisceral en-bloc resections. The outcomes are likely to be favorable and hence the results gratifying. Locally advanced rectal cancers require exenterative pelvic surgery which carries higher morbidity and mortality. In addition, rectal cancers are biologically more aggressive. Hence exenterative pelvic surgeries are to be done more selectively with guarded disease out come for T4 rectal tumors. Future improvements in chemotherapeutic agents and radiation techniques could make down staging of these malignancies a reality not only in terms of operability but in improving overall survival.

## List of abbreviations used

**US: **Ultrasound

**CEA: **Carcinoembryonic antigen

**CT: **Computerized tomogram

**5FU: **5-Fluoro-uracil

**MRI: **Magnetic Resonance Imaging

**IORT: **Intraoperative Radiotherapy

**DFS: **Disease free survival

**OS: **Overall survival

## Competing interests

The author(s) declare that they have no competing interests.

## Authors' contributions

**KH: **Was the principal treating surgeon apart from designing and conceptualizing the article.

**YVN: **Was the assistant in the procedures and made the preliminary draft of the article.

**SN: **Planned and administered adjuvant radiation.
